# Nanoporous carbon materials with enhanced supercapacitance performance and non-aromatic chemical sensing with C_1_/C_2_ alcohol discrimination

**DOI:** 10.1080/14686996.2016.1219971

**Published:** 2016-09-01

**Authors:** Lok Kumar Shrestha, Laxmi Adhikari, Rekha Goswami Shrestha, Mandira Pradhananga Adhikari, Rina Adhikari, Jonathan P. Hill, Raja Ram Pradhananga, Katsuhiko Ariga

**Affiliations:** ^a^ International Center for Materials Nanoarchitectonics (WPI-MANA), National Institute for Materials Science (NIMS), 1-1 Namiki, Ibaraki Tsukuba, Japan; ^b^ Central Department of Chemistry, Tribhuvan University, Kirtipur, Kathmandu, Nepal

**Keywords:** Nanoporous carbon materials, electrochemical properties, vapor sensing, non-aromatic chemical sensing, alcohol discrimination, 60 New topics/Others, 102 Porous / Nanoporous / Nanostructured materials, 104 Carbon and related materials, 208 Sensors and actuators

## Abstract

We have investigated the textural properties, electrochemical supercapacitances and vapor sensing performances of bamboo-derived nanoporous carbon materials (NCM). Bamboo, an abundant natural biomaterial, was chemically activated with phosphoric acid at 400 °C and the effect of impregnation ratio of phosphoric acid on the textural properties and electrochemical performances was systematically investigated. Fourier transform-infrared (FTIR) spectroscopy confirmed the presence of various oxygen-containing surface functional groups (i.e. carboxyl, carboxylate, carbonyl and phenolic groups) in NCM. The prepared NCM are amorphous in nature and contain hierarchical micropores and mesopores. Surface areas and pore volumes were found in the range 218–1431 m^2^ g^−1^ and 0.26–1.26 cm^3^ g^−1^, respectively, and could be controlled by adjusting the impregnation ratio of phosphoric acid and bamboo cane powder. NCM exhibited electrical double-layer supercapacitor behavior giving a high specific capacitance of *c*.256 F g^−1^ at a scan rate of 5 mV s^−1^ together with high cyclic stability with capacitance retention of about 92.6% after 1000 cycles. Furthermore, NCM exhibited excellent vapor sensing performance with high sensitivity for non-aromatic chemicals such as acetic acid. The system would be useful to discriminate C_1_ and C_2_ alcohol (methanol and ethanol).

## Graphical abstract

**Figure:**
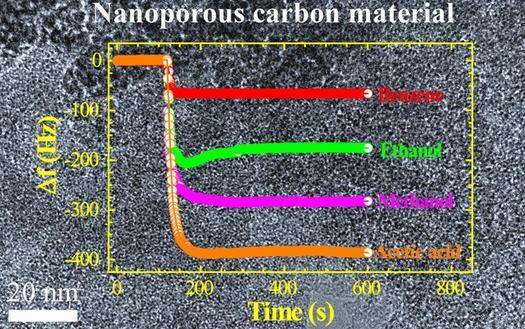

## 1. Introduction

Nanocarbons made from fullerenes,[1–3] carbon nanotubes,[4,5] graphenes,[6,7] and other advanced nanomaterials [8–10] have received much attention in various fields of application. In addition to such elite nanocarbon materials, nanoporous carbons with regular and non-regular pores of nanometer-level diameters, synthesized from naturally abundant materials have important practical uses.[11–13] These nanoporous carbon materials (NCM) have various applications including adsorbents, sensors and capacitors.[14–17] Especially desirable is fabrication of nanocarbon materials and devices from inexpensive natural sources, such as bamboo residue.

NCM can be prepared by physical and/or chemical activation processing of carbonaceous materials such as lignite, coal, peat, and wood. For physical activation, a precursor material is heated at high temperatures (800–1100 °C) in steam, carbon dioxide, nitrogen or air. This method generally suffers from low yield and surface areas of the physically activated NCM are low (around 1000 m^2^ g^−1^). For chemical activation, a precursor material is treated with dehydrating agents such as phosphoric acid (H_3_PO_4_), sodium hydroxide (NaOH), potassium hydroxide (KOH), sulfuric acid (H_2_SO_4_), potassium chloride (KCl), calcium chloride (CaCl_2_) or zinc chloride (ZnCl_2_), prior to carbonization at relatively low temperatures. Activating agents promote the pyrolytic decomposition of lignocellulosic materials through depolymerization and dehydration of their constituent biopolymers.[18–21] Chemical activation is preferred over physical activation because it leads to enhanced porosity in the products and consumes less energy. Specific surface areas of chemically activated NCM are much greater than those of physically activated carbons and porosity can be controlled by activation temperature, time, and impregnation ratio of the activating agents.

NCM can be prepared from various high-carbon-content lignocellulose precursors, with those that are generally abundant, renewable, inexpensive, and environmentally friendly being preferred. In recent years, extensive efforts have been made to recycle agro-waste materials in the fabrication of NCM.[22–26] Recently, Joshi et al. [27] prepared NCM from Lapsi (*Choerospondias axillaris*) seed, a waste material, using ZnCl_2_ activation. Phosphoric acid activation of corncob agro-waste also gave a high surface area NCM with excellent electrochemical supercapacitive performance.[28] Very recently, Li et al. [29] reported activated carbons with exceptionally high surface area (3931 m^2^ g^−1^) by the KOH activation of pine cone shell at 800 °C.

In this contribution, we report two major results: (i) the synthesis of NCM from a highly naturally abundant source, bamboo, under a low temperature chemical process; (ii) the functions of the prepared NCM, especially for unusual non-aromatic chemical sensors. Bamboo cane powder was chemically activated with phosphoric acid at 400 °C and the effect of impregnation ratio of phosphoric acid on the surface areas, pore volumes, and electrochemical supercapacitive performances was systematically investigated. Furthermore, vapor sensing properties of selected NCM were also studied using the quartz crystal microbalance (QCM) technique. Although most NCM exhibit sensing preferences to aromatic compounds due to well-developed *sp* ^2^ carbon structures,[30–33] the newly prepared NCM with suppressed *sp* ^2^ nature showed unusual specificity to a small non-aromatic molecules such as acetic acid. Even the difficult discrimination between methanol and ethanol becomes possible with this strategy.

## 2. Experimental details

### 2.1. Materials

Bamboo cane, a precursor material of activated carbon, was collected from the local forest. Orthophosphoric acid (H_3_PO_4_: 88% w/w), sulfuric acid (H_2_SO_4_), ethanol, propanol and ethylbenzene were purchased from Nacalai Tesque Inc. Kyoto, Japan. Methanol, butanol and carbon tetrachloride were obtained from Wako Pure Chemical Industries, Tokyo, Japan. Benzene and toluene were obtained from Tokyo Chemical Industries Co. Ltd., Tokyo, Japan. All chemicals were used as received. Distilled water was used to prepare solutions.

### 2.2. Methods

#### 2.2.1. Preparation of NCM

The bamboo cane was washed with distilled water and dried in a hot air oven at 120 °C for 24 h. The dried bamboo cane was then cut into pieces and ground in an electric grinder. The resulting bamboo powder (BP) was then chemically activated using phosphoric acid at different mixing ratios: H_3_PO_4_:BP = 0.1:1, 0.4:1, 0.7:1, 1:1, 1.5:1, and 2:1 at 400 °C under a continuous flow of nitrogen gas (100 cc min^−1^). The carbonized products were allowed to cool to room temperature then washed several times with distilled water to remove excess acid, and finally dried at 110 °C for 6 h in a hot air oven. The dried carbon samples were ground to powder and used for further studies. The products obtained are denoted as NCM_*x*, where *x* (= 0.1, 0.4, 0.7, 1.0, 1.5, and 2.0) indicates the impregnation ratio of phosphoric acid.

#### 2.2.2. Characterization

NCM_*x* samples were characterized by various techniques. Surface functional groups were detected by using Fourier transform-infrared (FTIR) spectroscopy. FTIR spectra were recorded between 4000 and 400 cm^−1^ on a Nicolet 4700 FTIR (Thermo Scientific, Minnesota, USA). Surface chemistry of NCM_*x* (chemical states of elements) was studied by X-ray photoelectron spectroscopy (XPS). XPS measurements were performed on a Theta Probe spectrometer (Thermo Electron Corporation, Waltham, Massachusetts, USA) using monochromated Al-K_α_ radiation (photon energy 15 keV). High resolution core level C 1s, and O 1s spectra were recorded in 0.05 eV steps. A built-in electronic charge neutralizing electron flood gun was used to prevent sample charging. Powder X-ray diffraction (pXRD) patterns were recorded using a Rigaku RINT Ultima III X-ray diffractometer (Rigaku, Tokyo, Japan) operated at 40 kV and 40 mA with Cu-K_α_ radiation. Raman scattering measurements were carried out on Jobin-Yvon T64000 Raman spectrometer, Kyoto, Japan. Samples were excited using a green laser at 514.5 nm and 0.5 mW power. Samples were prepared on clean glass substrates. Surface morphology was studied using scanning electron microscopy (SEM). Samples for SEM were prepared on a cleaned silicon wafer substrate and images were taken at an accelerating voltage of 10 kV using a Hitachi S-4800 FE-SEM (Hitachi Co. Ltd, Tokyo, Japan). Structure and morphology was also investigated using transmission electron microscopy (TEM). High resolution-TEM (HR-TEM) images and electron diffraction patterns were recorded from the smallest components of the sample using a JEOL Model JEM-2100F (Tokyo, Japan) transmission electron microscope operated at 200 kV. TEM samples were prepared by placing a drop of a suspension of NCM_*x* in isopropanol on a carbon coated copper grid followed by drying under reduced pressure for 24 h prior to TEM observations.

#### 2.2.3. Nitrogen adsorption–desorption isotherm

Specific surface area and pore volume were investigated by recording N_2_ adsorption-desorption isotherms on an automatic adsorption instrument (Quantachrome Instrument, Autosorb-1, Boynton Beach, Florida USA). Samples (~25 mg) were degassed for 24 h at 120 °C prior to the measurement. The adsorption–desorption isotherms were recorded at liquid nitrogen temperature (77 K).

#### 2.2.4. Electrochemical measurements

The electrochemical capacitive performances of NCM_*x* were evaluated by cyclic voltammetry (CV) and chronopotentiometry techniques. Cyclic voltammograms (CV) were recorded in a three-electrode system in 1 M H_2_SO_4_ solution at 25 °C versus Ag/AgCl reference electrode in the potential range of 0 to 0.8 V. A bare glassy carbon electrode (GCE) used as working electrode was mirror polished with Al_2_O_3_ slurry and cleaned with double-distilled water and sonicated in acetone for 5 min. NCM sample (2 mg) was dispersed in 2 ml of ethanol (1 mg ml^−1^) and the mixture was sonicated for 60 min in a sonication bath. The dispersion of NCM (3 μl) was added onto the GCE surface and dried at 60 °C for 6 h. After the solvent was evaporated, 5 μl of Nafion solution (5%) was added as binder on the surface of the GCE and dried at 60 °C for 6 h. A platinum wire was used as a counter electrode and Ag/AgCl as the reference electrode. The cyclic voltammetry response and chronopotentiometry were performed on a CH instruments model: (CHI 850D Work station (USA)). In chronopotentiometry measurements charge-discharge (CD) curves were also recorded at different current densities (1–10 A g^−1^). For the cyclic stability test, CD curves were recorded up to 1000 cycles. Specific capacitance (C_s_) from CV curve was calculated using the following equation:

(1) $$C_{s}=\frac{1}{mv(V_{f}-V_{i})}\int_{V_{i}}^{V_{f}}I\left(V\right)\text{d}V$$

where *C*_s_ is the specific capacitance, *m* is the mass of the active electrode material, *v* is scan rate, *V*_f_ and *V*_i_ are the integration limits of the voltammetry curve, and *I*(*V*) represents the current response, respectively. Specific capacitance was also calculated from CD curves using the following equation:

(2) $$C_{s}=\frac{It}{\Delta V\times m}$$

Here, *I*, *t*, Δ*V*, and *m* are discharge current (A), the discharge time (s), potential window, and mass of the active material on the electrode, respectively.

#### 2.2.5. Quartz crystal microbalance (QCM) test

Adsorption of solvent vapors on NCM_1 was studied using quartz crystal microbalance (QCM) technique. The QCM electrode was coated with NCM and frequency was measured during exposure to solvent vapor at room temperature (25 °C). The change in mass *m* (g cm^−1^) of the carbon material deposited on the surface of the QCM electrode is related to the change in the oscillating frequency of the quartz electrode. For the preparation of QCM electrode, NCM_1 was dispersed in water (2 mg ml^−1^) by sonication for 1 h. The suspension of NCM_1 (3 μl) was then drop casted onto the gold electrode and dried for 24 h in a vacuum. The QCM electrode was then fixed in the QCM instrument and frequency was recorded upon exposing different solvent vapors.

## 3. Results and discussion

**Figure 1.**
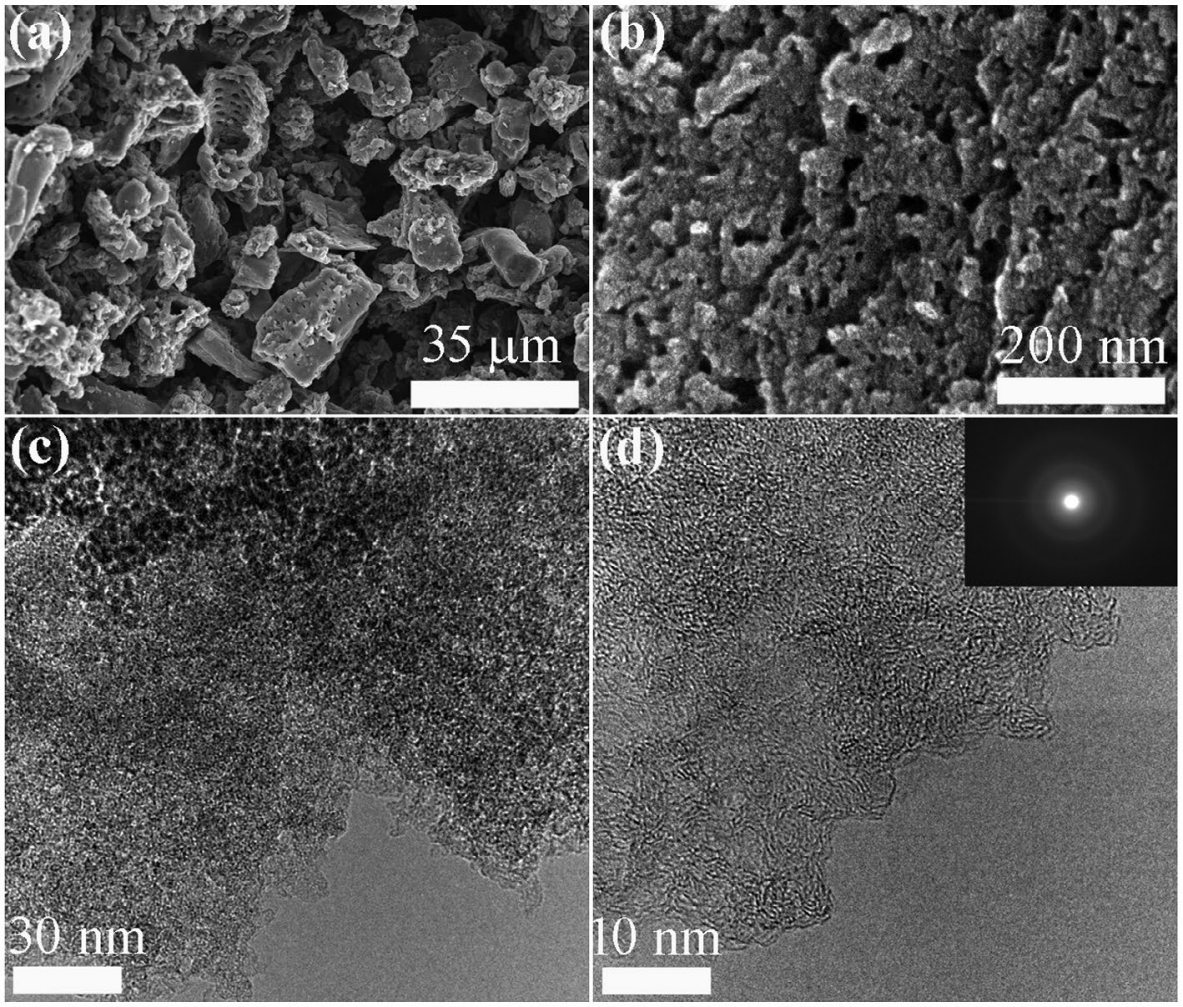
Electron microscopy observation of NCM_1 as a typical example: (a, b) SEM; (c) TEM; (d) HR-TEM. Inset of panel (d) shows a selected area electron diffraction (SAED) pattern.

Figure 1 shows typical SEM, TEM, and HR-TEM images of NCM_1. The SEM image (Figure 1(a)) reveals fine granules of carbon with irregular particle size. The surfaces of the carbon particles contain numerous mesopores and macropores. The mesoporous structure with pore size in the range of 10–50 nm can be seen in the high magnification SEM image (Figure 1(b)). From SEM observations, it appears that mesoporosity seems to increase with increases in the impregnation ratio of phosphoric acid from 0.1 to 1 and then remains almost unchanged (Figure S1 in Supporting Information) revealing that higher acid concentrations creates more pores. The highly porous net like surface structure can also be observed by TEM (Figure 1(c)). Amorphous carbon structure with partial random graphitic layer, common to activated carbon, can be seen in HR-TEM image (Figures 1(d) and S2).

**Figure 2.**
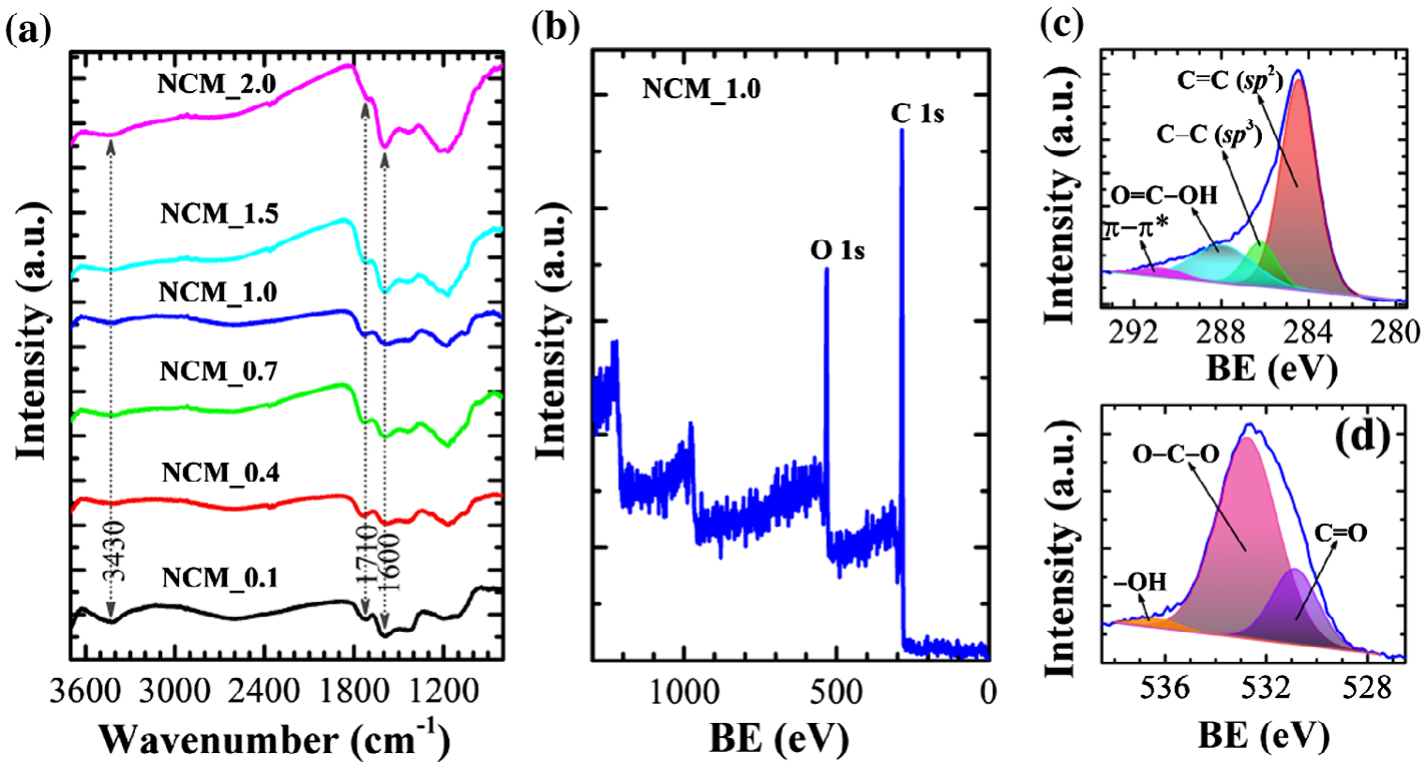
(a) FTIR spectra and (b) XPS survey spectra of NCM_*x* at different *x*. (c) XPS C 1s core level spectrum of NCM_1, and (d) the corresponding O 1s spectrum. BE stands for binding energy.

FTIR spectra of NCM_*x* (*x* = 0.1, 0.4, 0.7, 1.0, 1.5, and 2.0) indicate the presence of several oxygen-containing surface functional groups including –OH, C=O, COOH, ether, phenol and lactones (Figure 2(a)). The major absorption bands appear at 3430 cm^−1^ (O–H stretching vibration of hydroxyl groups), 1710 cm^−1^ (C=O stretching of carboxylic acid groups), and 1600 cm^−1^ (C=C stretching in aromatic compounds). Furthermore, a weak absorption band in the range 1000–1300 cm^−1^ (C–O stretching) is also present.[34] Note that the broad band around 3440 cm^−1^ becomes less intense especially at higher impregnation ratios due to the elimination of -OH functionality. The presence of surface functional groups was further confirmed by XPS. XPS survey spectra (Figure 2(b)) clearly display core level peaks for carbon (C 1s: 80.2 atomic %) and oxygen (O 1s: 19.8 atomic %). The XPS C 1s core level peak of NCM_1 could be deconvoluted into four peaks at 284.4 (C=C; *sp* ^2^), 286.1 (C–C; *sp* ^3^), 287.9 (O–C=O), and 290.7 eV (π–π* shake up) (Figure 2(c)). The XPS O 1s core level spectrum of NCM_1 could be deconvoluted into three peaks at 530.8 (C=O), 532.71 (O–C–O) and 536.2 eV (–OH). Thus XPS data demonstrates the hetero-carbon components with oxygen-containing functional groups [34, 35].

**Figure 3.**
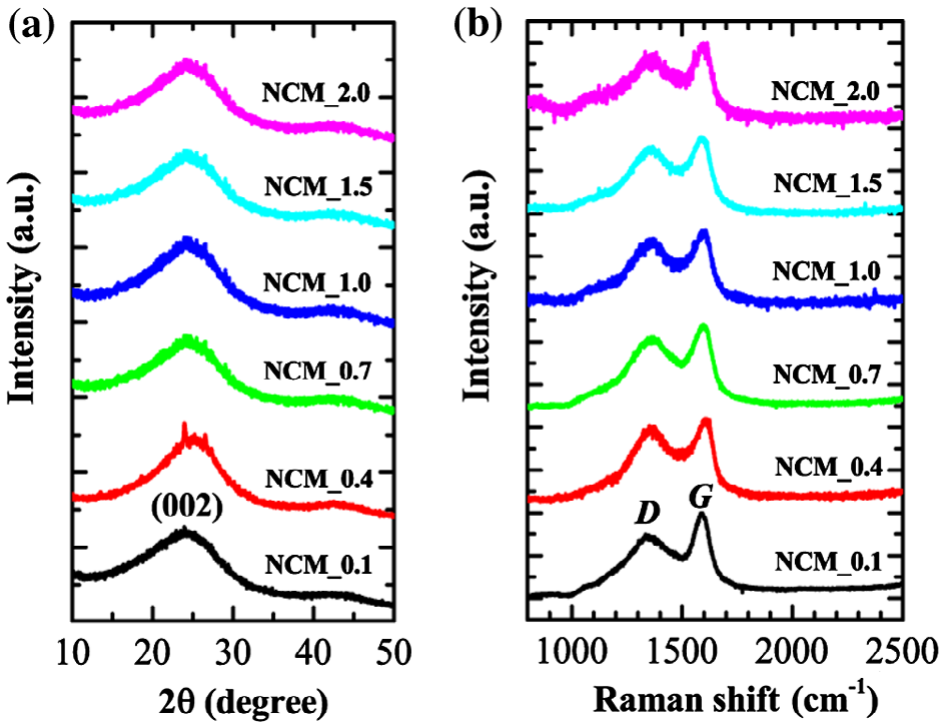
(a) XRD patterns; and (b) Raman scattering spectra of NCM_ *x* (*x* = 0.1, 0.4, 0.7, 1.0, 1.5, and 2.0).

The XRD patterns and Raman scattering spectra of NCM_*x* (*x* = 0.1, 0.4, 0.7, 1.0, 1.5, and 2.0) are summarized in Figure 3. XRD patterns of NCM mostly contain two broad peaks at diffraction angles ~ 25 ° and 43 ° (weak) (Figure 3(a)), which is typical of amorphous carbon and can be attributed to the (002) and (100) planes of graphitic clusters.[36,37] The broad peaks observed may also indicate that the graphitic clusters in the NCM are small and, as revealed by HR-TEM image (Figure 1(d)), the ordered graphene layers are not fully developed. Small XRD peaks particularly in NCM_0.4 originate from inorganic impurities. Note that in case of phosphoric acid activation, phosphate and polyphosphate species are incorporated in the carbon matrix through C–O–P bonds; as a result, all the phosphorus would not be removed with washing. Furthermore, lignocellulosic materials contain several metal impurities. The impurity peaks might have come from metal phosphates. Raman scattering spectra of NCM samples contain two broad bands typical of amorphous carbons approximately at 1350 (*D*) and 1597 cm^−1^ (*G*).[38] It is well known that the *D* band (defect induced band) is caused by the disordered structure of graphene while the *G* band is due to the E_2 g_ mode and arises from C–C bond stretching in graphitic materials and is common to all *sp* ^2^ carbon systems. We have investigated the effect of impregnation ratio of phosphoric acid on the degree of graphitization of the NCM prepared by estimating the relative intensity of the *G* and *D* bands (I_G_/I_D_).[39] I_G_/I_D_ is found in the range 1.07 to 1.27 (partially graphitized) and does not follow any trend based on the impregnation ratio of phosphoric acid.

**Figure 4.**
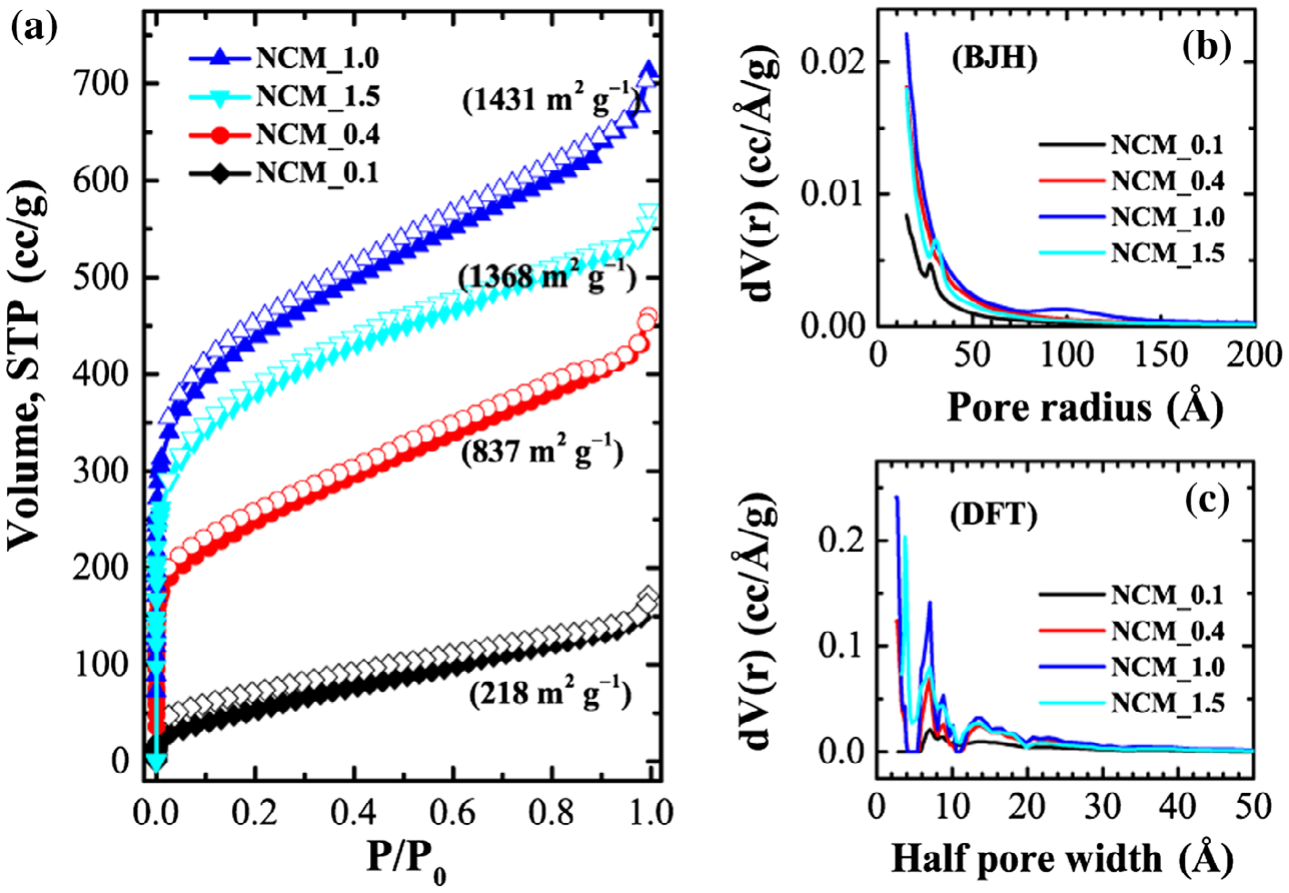
(a) Nitrogen adsorption–desorption isotherms of NCM_ *x* (*x* = 0.1, 0.4, 0.7, 1.0, 1.5, and 2.0); and corresponding pore size distributions as obtained from (b) BJH and (c) DFT methods.

Figure 4 exhibits nitrogen sorption isotherms and the corresponding pore size distributions obtained by Barrett-Joyner-Halenda (BJH) and density functional theory (DFT) for selected samples. Nitrogen uptake is strongly dependent on the sample, demonstrating that the degree of phosphoric acid impregnation plays an important role in determining the surface textural properties (surface area and pore volume) of NCM. Sorption isotherms are essentially mixed Type-I/Type-IV so that nitrogen uptake increases significantly at lower relative pressure with a hysteresis loop at higher relative pressures, indicating the presence of both micro- and mesopore structures in the prepared NCM. High nitrogen adsorption at low relative pressure is attributed to the filling of micropores while the hysteresis phenomenon is due to capillary condensation occurring in the mesopores. Note that the volume of nitrogen uptake increases with increasing phosphoric acid impregnation ratio up to *x* = 1 and then decreases. This suggests that increasing phosphoric acid impregnation ratio creates additional pores (meso and micropores) on the surface of NCM leading to an optimization of surface area. Further increases in impregnation ratio lead to a decrease in surface area perhaps caused by the destruction of micro- and mesopores at high acid concentrations. Brunauer-Emmett-Teller (BET) surface areas are *c*.218 m^2^ g^−1^ (NCM_0.1), 837 m^2^ g^−1^ (NCM_0.4), 1243 m^2^ g^−1^ (NCM_0.7), 1431 m^2^ g^−1^ (NCM_1), 1368 m^2^ g^−1^ (NCM_1.5), and 1187 m^2^ g^−1^ (NCM_2.0). Similarly, total pore volumes are ca. 0.26 cm^3^ g^−1^ (NCM_0.1), 0.71 cm^3^ g^−1^ (NCM_0.4), 0.91 cm^3^ g^−1^ (NCM_0.7), 1.10 cm^3^ g^−1^ (NCM_1), 0.88 cm^3^ g^−1^ (NCM_1.5), and 1.26 cm^3^ g^−1^ (NCM_2.0).

Note that bamboo is a lignocellulosic material containing hemicellulose, cellulose and lignin as the main components. During carbonization, these polymeric structures decompose and release most of the non-carbon elements such as hydrogen, oxygen and nitrogen in liquid or gas phase, leaving behind a rigid carbon skeleton. When precursor is mixed with phosphoric acid in chemical activation process, the reaction of lignocellulose with phosphoric acid begins. Phosphoric acid first attacks hemicellulose and lignin as cellulose is known to be more resistant to acid hydrolysis. The phosphoric acid also hydrolyzes glycosidic linkages in lignocellulose and cleaves aryl ether bonds in lignin. These reactions are accompanied by chemical transformations including dehydration, degradation, and condensation. As the phosphoric acid concentration increases, the aromatic condensation reactions also take place among the adjacent molecules, which result in the evolution of gaseous products from the hydroaromatic structure of carbonized char. Furthermore, the excess phosphoric acid will promote gasification of char and increase the total weight loss of carbon. During the phosphoric acid chemical activation process, the activating agent (phosphoric acid) gets intercalated in the internal structure of lignocellulosic materials and is responsible for porosity generation. It is known that removing the chemical activating agents in the carbonized sample by washing yields porosity in the carbon structure. Therefore, as the impregnation ratio of phosphoric acid increases, the volume filled by phosphoric acid and various polyphosphates increases, resulting in the large pore size and pore volume.[40–42]

**Figure 5.**
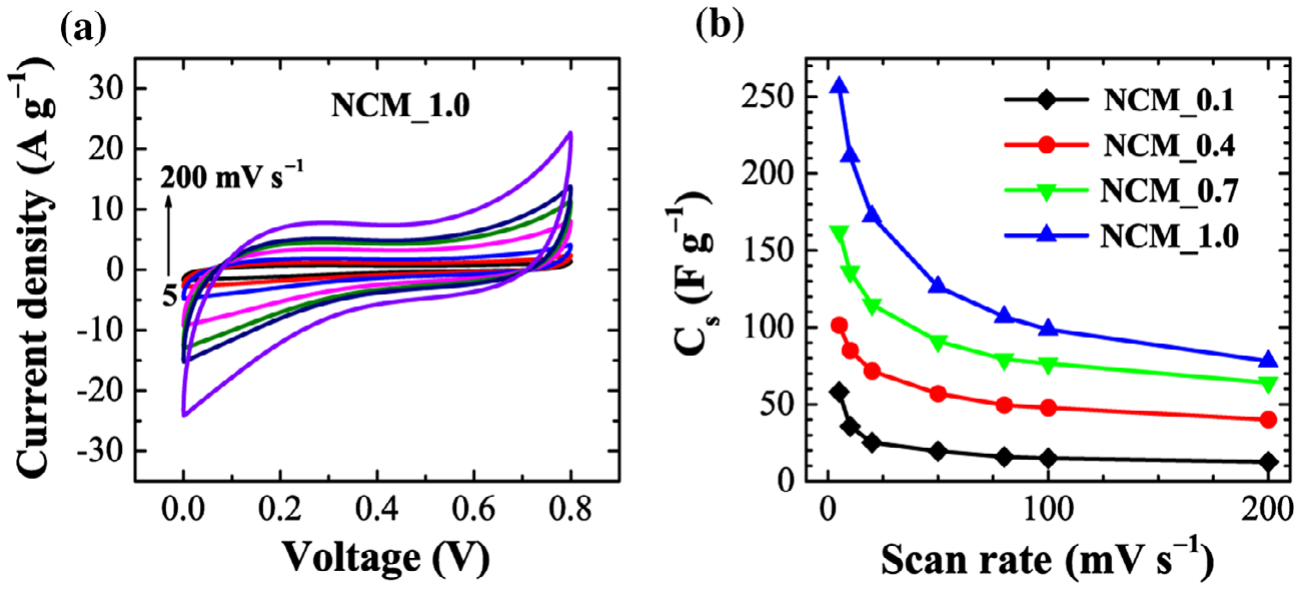
Electrochemical performance of NCM: (a) cyclic voltammograms of NCM _1 at different scan rates; and (b) the calculated specific capacitances for NCM_*x*, *x* = 0.1, 0.4, 0.7, and 1.0.

Considering the large surface area and large porosity of NCM, we have studied the electrochemical supercapacitive performance by using cyclic voltammetry (CV). Figure 5(a) shows the CV curves of NCM_1 at different scan rates (5, 10, 20, 50, 80, 100 and 200 mV s^−1^) recorded in the potential range of 0 to 0.8 V against an Ag/AgCl reference electrode in 1 M H_2_SO_4_ as an electrolyte. An electrode prepared using the other samples exhibited similar behavior. Note that NCM_1 shows a rapid current response to voltage at each end potential giving an approximately rectangular shaped CV curve, which would be ideal for electrical double layer capacitor applications.[43] It should be noted that a perfect rectangular shaped CV curve can only be found in an ideal supercapacitor. In real systems, electrolyte ion diffusion may be obstructed by migration force and polarized resistance is expected to be produced so that the CV curve deviates from the ideal rectangle. Furthermore, heteroatoms such as oxygen and nitrogen present in carbon may undergo Faradic redox reaction and the CV curves deviate from the rectangular shape. However, within the studied potential window, we did not observe such Faradic redox reactions from the oxygen heteroatom. As can be seen in Figure 5(a), the approximately rectangular shaped CV curve is retained even at higher scan rates (200 mV s^−1^), which is an indication of rapid electrolyte ion diffusion even at higher scan rates. The calculated specific capacitances (C_s_) for NCM_*x*, *x* = 0.1, 0.4, 0.7, and 1.0 presented in Figure 5(b) clearly demonstrate that C_s_ increases with *x* and that the highest C_s_ was obtained at *x* = 1.0 (NCM_1), which corresponds with the BET results. That is, larger surface area and pore volume lead to greater specific capacitances. C_s_ calculated from CV curves for NCM_1 are 256, 211, 172, 126, 106, 98, and 78 F g^−1^ at scan rates of 5, 10, 20, 50, 80, 100, and 200 mV s^−1^, respectively, revealing capacitance retention of about 30% even at higher scan rates (200 mV s^−1^).

**Figure 6.**
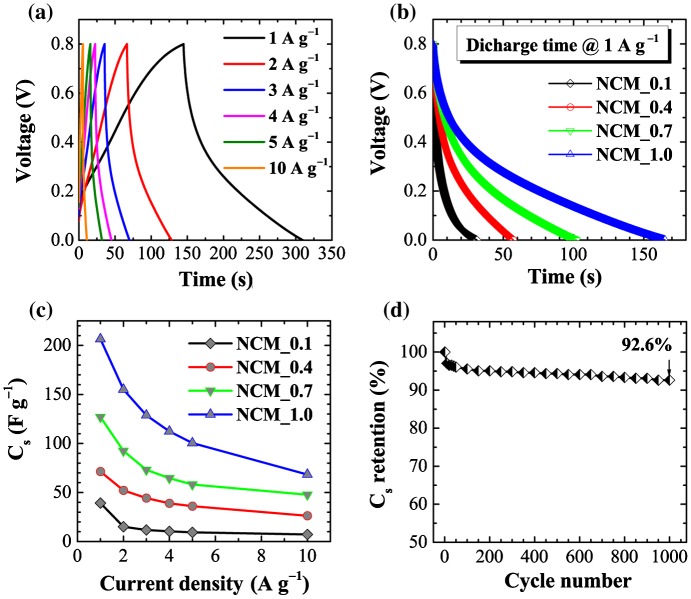
(a) Charge–discharge curves of NCM_1 at different current densities (1, 2, 3, 4, 5, and 10 Ag^−1^) in the potential range of 0 to 0.8 V as typical example; (b) comparison of discharge time for NCM_*x*, *x* = 0.1, 0.4, 0.7, and 1.0 at a constant current density of 1 A g^–1^; (c) calculated specific capacitances for NCM_*x*, *x* = 0.1, 0.4, 0.7, and 1.0; and (d) cyclic stability plot up to 1000 cycles.

Electrochemical performance was also studied by chronopotentiometry. Figure 6(a) shows galvanostatic charge–discharge curves for the NCM_1 sample at different current densities (1, 2, 3, 4, 5, and 10 A g^−1^) in the potential range 0 to 0.8 V. Charge-discharge curves again display the typical behavior of electrical-double layer capacitors: an almost triangular shaped CD curve with symmetric legs. An apparently linear decay of the discharge curve indicates well-balanced charge storage. Figure 6(b) compares the discharge times of different electrodes (NCM_*x*, *x* = 0.1, 0.4, 0.7, and 1.0) at a constant current density of 1 A g^−1^. The discharge time increases monotonously with *x*, demonstrating higher capacitive properties at higher *x*. The calculated C_s_ from the discharge curve versus current density is presented in Figure 6(c). As revealed by CV data, C_s_ increases with phosphoric acid impregnation ratio, giving maximum specific capacitance for NCM_1.0. The C_s_ are *c*.206, 155, 128, 112, 100, and 68 F g^−1^ at current densities of 1, 2, 3, 4, 5, and 10 A g^−1^, respectively. In a cyclic stability test, we found that the NCM_1 electrode shows excellent cyclic stability (Figure 6(d)) with specific capacitance retention of about 93% after 1000 cycles. These results show that NCM_1 meets the criteria of high stability and good cyclic stability for use in electrical double layer supercapacitors.[44]

**Figure 7.**
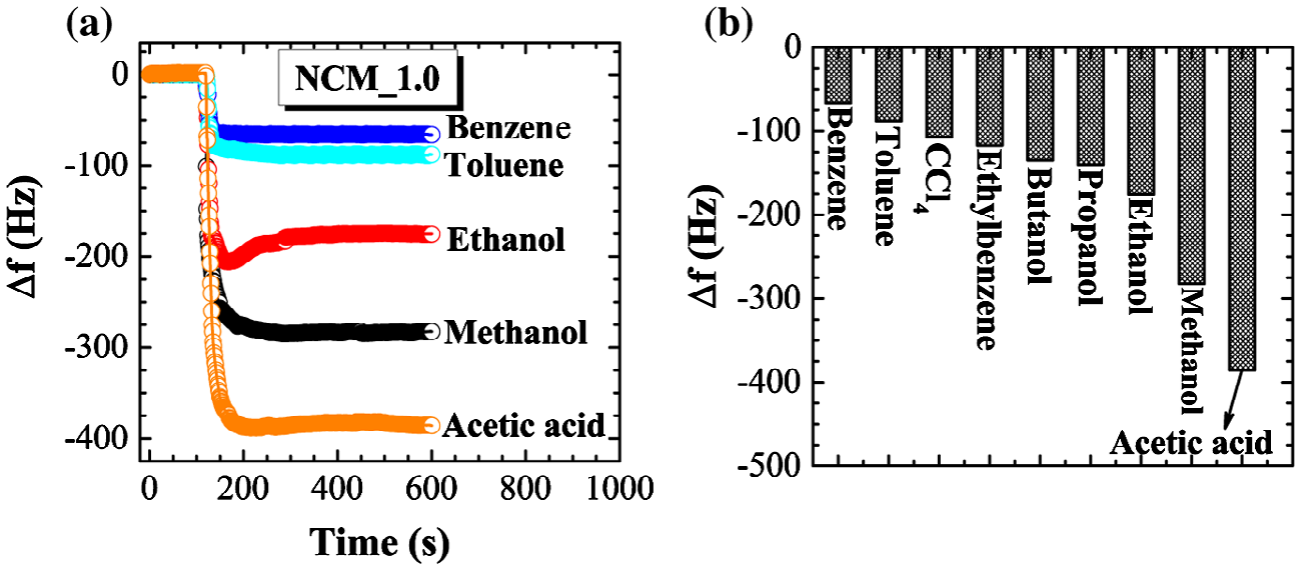
(a) QCM frequency shifts upon exposure to methanol, ethanol, benzene, toluene, ethylbenzene, and acetic acid to the NCM_1 coated QCM electrode; and (b) summary of sensing performance.

Adsorption of toxic solvent vapors on nanoporous carbons has received considerable recent attention. Previous investigations have highlighted the necessity of a well-designed host architecture for effective adsorption.[45] It has been found that porous materials with high surface area and large pore volume offer better vapor sensing performance.[46] Judging from the excellent surface and structural properties, we supposed that our NCM_1 could be a suitable candidate for vapor sensing of volatile organic solvents. Therefore, we determined the vapor sensing performance for various organic solvents using the quartz crystal microbalance (QCM) technique. Figure 7(a) shows the time dependencies of frequency shifts for the QCM electrode prepared using NCM_1 upon exposure to different solvent vapors (methanol, ethanol, benzene, toluene, and acetic acid) as typical example. Note that QCM frequency shifts are very rapid upon exposure of the QCM electrode to these solvents. Furthermore, the frequency shift depends largely on the nature of the solvent. As seen in some cases, over-adsorption behaviors were observed during the initial adsorption process, which indicates QCM frequency changes during the adsorption process can be affected by complicated factors including actual over-adsorption of the guest and mechanical contact of the guest on the surface. Therefore, reliable comparisons of the guest selectivity were made by frequency changes after the adsorption equilibrium (longer adsorption time). Frequency shift caused by exposure to aromatic solvent vapor benzene (66 Hz) is much lower than the shift due to exposure to acetic acid (385 Hz), methanol (282 Hz) or ethanol (175 Hz), indicating greater sensitivity of NCM_1 material for acetic acid. The higher sensitivity for non-aromatic solvent vapors such as methanol and ethanol compared to the aromatic benzene and toluene vapors might be caused due to the presence of oxygen heteroatoms present in the NCM_1. The interaction between oxygen containing surface functional groups (–OH, C=O, and COOH) and alcohol vapors may promote their adsorption on the NCM_1. In the tested cases, the oxygen atom and the hydrogen atom next to that oxygen atom in the guest gas molecules are interactive sites for these functional groups. Because methanol has higher density of the interactive site (OH per one carbon) than ethanol (OH per two carbons), the former probably showed higher sensitivity than the latter. On the other hand, it seems that adsorption of aromatic solvent molecules into the nanoporous carbon structure is limited due to the lack of a well-developed graphitic microstructure. In case of graphitic nanoporous carbons, adsorption of aromatic solvent vapors is increased due to strong *π*–*π* interactions between solvent molecules and the *sp* ^2^-bonded graphitic carbon framework.

The sensitivity of sensing among the solvent vapors studied decreases in the following order: acetic acid > methanol > ethanol > propanol > butanol > ethylbenzene > carbon tetrachloride > toluene > benzene (Figure 7(b)). Because most sensors with NCM have higher preference for aromatic substances, the current examples become rather rare non-aromatic sensitive chemical sensors. In addition, we have to pay attention to significant differences in sensitivities between methanol and ethanol. Discrimination of these two essential C_1_ and C_2_ alcohols is not always easy,[47] although there is a great demand for separation and selective sensing of these two chemicals from viewpoints of petroleum and food industries. Therefore, the present approach using naturally abundant carbon sources may find practical industrial applications.

## 4. Summary

In summary, we have prepared high surface area NCM by chemical activation of bamboo cane powder with phosphoric acid at 400 °C. The effect of phosphoric acid impregnation ratio on the surface textural properties (surface area and pore volume), electrochemical supercapacitive and vapor sensing performance have been systematically studied. We have found that both the surface area and pore volume of bamboo derived nanoporous carbon increase with phosphoric acid concentration up to a certain point and then decrease slightly. Surface functionality of the materials remains similar at all acid concentrations studied. Surface area was improved from *c*.218 to 1431 m^2^ g^−1^ by increasing the impregnation ratio of phosphoric acid in bamboo cane powder from 0.1 to 1.0. Similarly, pore volume could be increased from 0.26 to 1.26 cm^3^ g^−1^. The prepared NCM showed excellent electrical double-layer supercapacitor performance, giving specific capacitance *c*.256 F g^−1^ at a scan rate of 5 mV s^−1^, which is much higher than the specific capacitance reported for commercially available activated carbons. Additionally, we have also observed high cyclic stability with capacitance retention of about 92.6% even after 1000 cycles. These observations suggest the possible use of our NCM in energy storage device fabrication. Furthermore, our NCM showed excellent solvent vapor sensing performance with high sensitivity for acetic acid and fine discrimination between methanol and ethanol.

## Disclosure statement

No potential conflict of interest was reported by the authors.

## Funding

This work was funded by the World Premier International Research Initiative (WPI Initiative), MEXT, Japan.

## Supplemental data

Supplemental data for this article can be accessed here. http://dx.doi.org/10.1080/14686996.2016.1219971

## Supplementary Material

suppl_data.zip
