# Supplementary material for: Nanoporous carbon materials with enhanced supercapacitance performance and non-aromatic chemical sensing with C1/C2 alcohol discrimination
Source: Sci Technol Adv Mater. 2016 Sep 1;17(1):483–92. doi: 10.1080/14686996.2016.1219971 (PMC5101920; doi:10.1080/14686996.2016.1219971)
Supplement: suppl_data.zip [file tsta_a_1219971_sm8365.zip › suppl_data/STAM-2016-0097R2-ESI.pdf]

Electronic Supporting Information (ESI)

Nanoporous carbon materials with enhanced supercapacitance performance and non-aromatic chemical sensing with C_1/C_2 alcohol discrimination

Lok Kumar Shrestha,[1]* Laxmi Adhikari,[2] Rekha Goswami Shrestha,[1] Mandira Pradhananga Adhikari,[2] Rina Adhikari,[2] Jonathan P. Hill,[1] Raja Ram Pradhananga,[2] and Katsuhiko Ariga[1]*

1. *International Center for Materials Nanoarchitectonics (WPI-MANA), National Institute for Materials Science (NIMS), 1-1 Namiki, Ibaraki Tsukuba, 305-0044, Japan*
2. *Central Department of Chemistry, Tribhuvan University, Kirtipur, Kathmandu, 44613, Nepal.*

*Contacts:

Dr. Lok Kumar Shrestha
SHRESTHA.Lokkumar@nims.go.jp
Tel: +81-29 860 4809
Fax: +81-29 860 4832

Prof. Dr. Katsuhiko Ariga
ARIGA.Katsuhiko@nims.go.jp
Tel: +81-29 860 4597
Fax: +81-29 860 4832

1. SEM observations

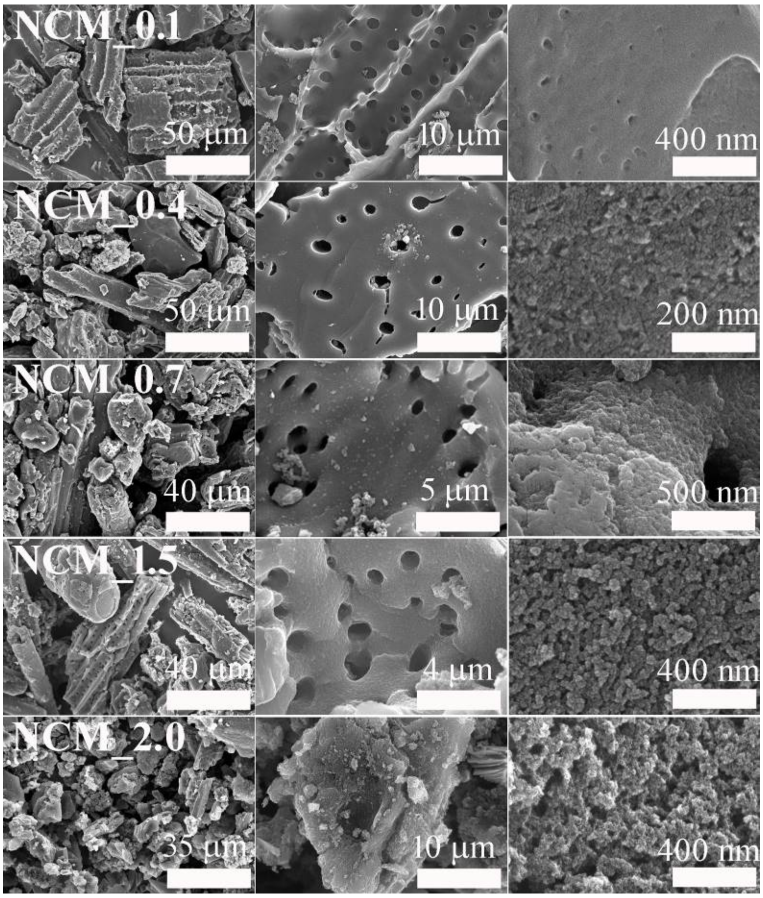

Figure S1: SEM images of NCM_*x* (*x* = 0.1, 0.4, 0.7, 1.5, and 2.0) at different magnifications. The increase of mesoporosity of the material can be evidenced by high resolution SEM images on the right.

2. TEM observations

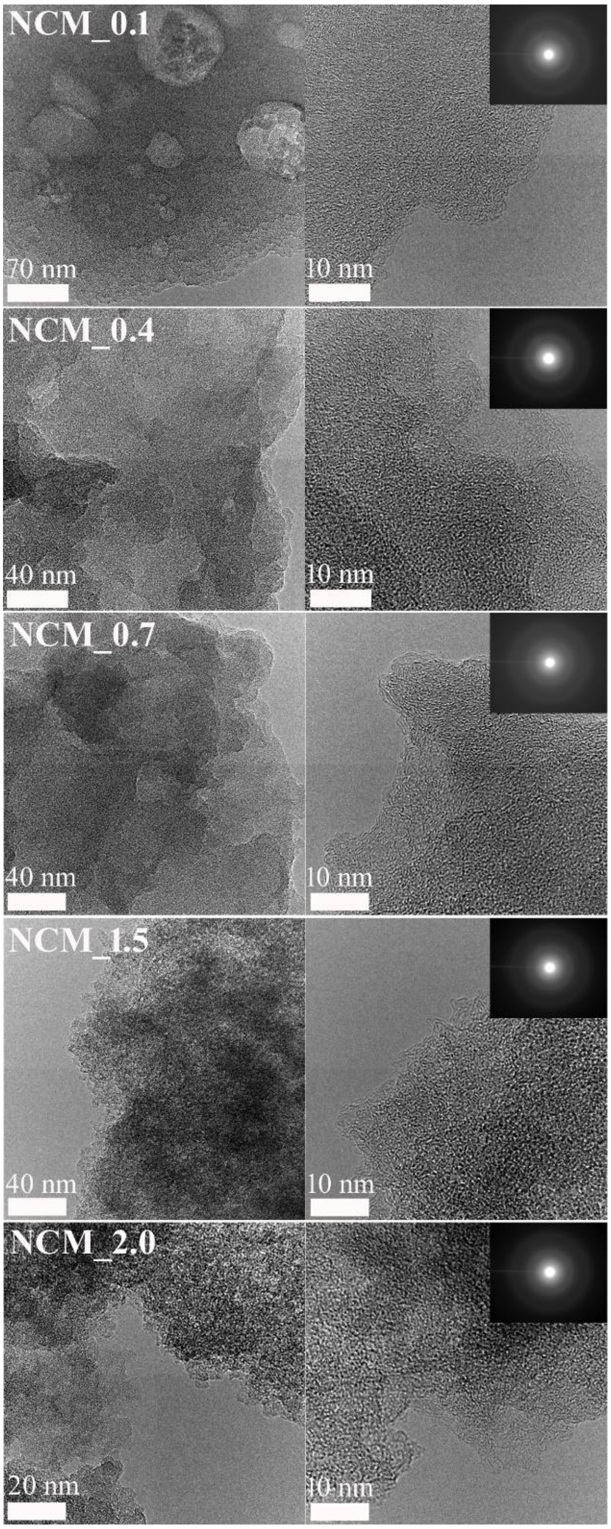

Figure S2: TEM (left) and HR-TEM (right) images of NCM_*x* (*x* = 0.1, 0.4, 0.7, 1.5, and 2.0). Insets of HR-TEM images show selected area electron diffraction (SAED) pattern.
