# Supplementary figures and images for: Nanoporous carbon materials with enhanced supercapacitance performance and non-aromatic chemical sensing with C1/C2 alcohol discrimination
Source: Sci Technol Adv Mater. 2016 Sep 1;17(1):483–92. doi: 10.1080/14686996.2016.1219971 (PMC5101920; doi:10.1080/14686996.2016.1219971)

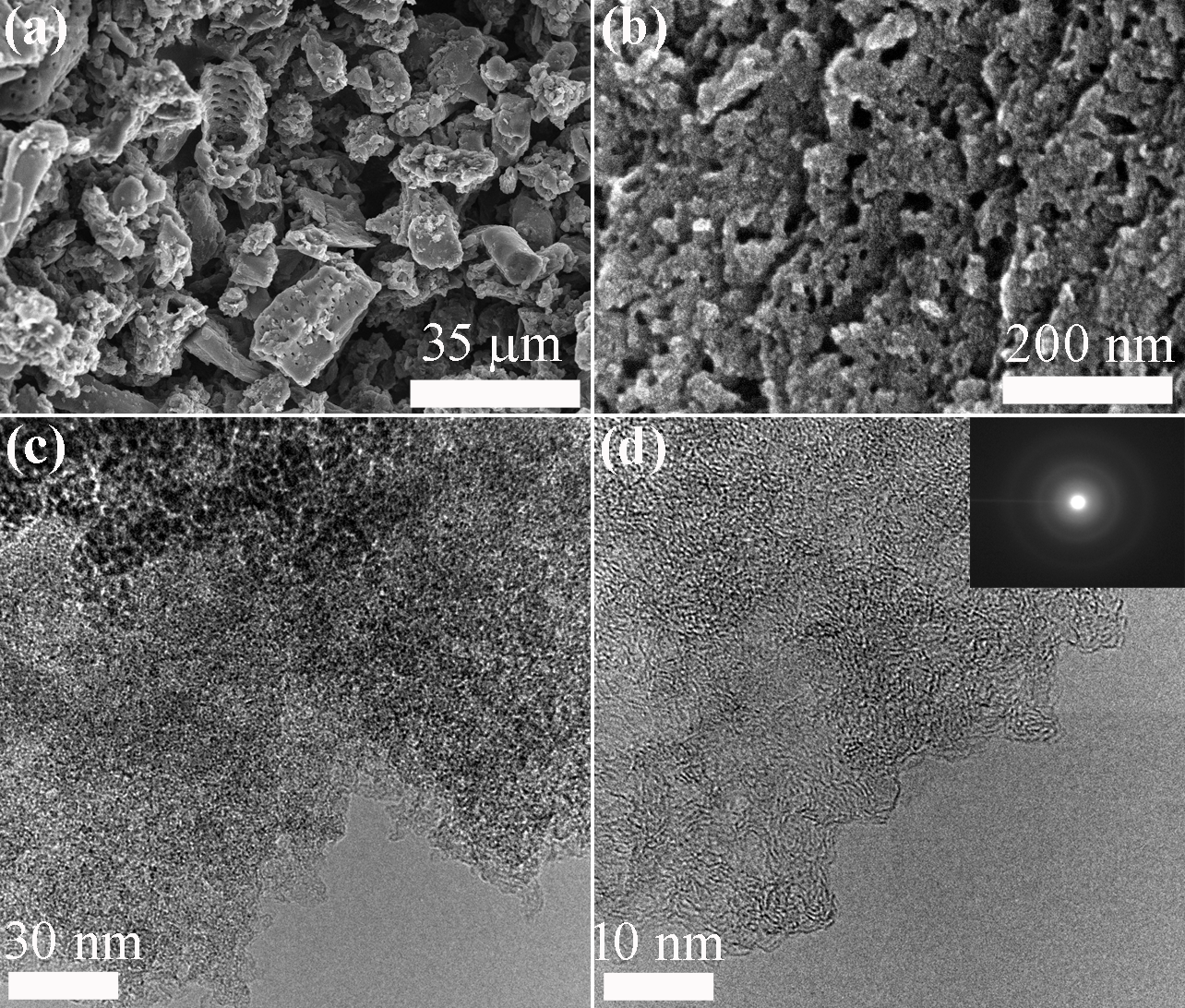

Supplement: suppl_data.zip [file tsta_a_1219971_sm8365.zip › suppl_data/STAM-2016-0097R2-Fig1.tif]
